# Supplementary material for: Sparstolonin B Suppresses Proliferation and Modulates Toll-like Receptor Signaling and Inflammatory Pathways in Human Colorectal Cancer Cells
Source: Pharmaceuticals (Basel). 2025 Feb 21;18(3):300. doi: 10.3390/ph18030300 (PMC11945018; doi:10.3390/ph18030300)
Supplement: Supplementary file 1 [file pharmaceuticals-18-00300-s001.zip › pharmaceuticals-3478104-supplementary.pdf]

Supplementary Table S1. Primers and probes used for RT-PCR.

GENE	OLIGO NAME	SEQUENCE*	5' DYE	3' DYE	GENEBANK / NO	BRAND	ORIGIN
	HTLR2YP1	CAAAGACACACACACAGAAATG	-	-		PROBSYNTESIS	TÜRKİYE
TLR2	TLR2KKP2	GCTTATCACTGGCTTGCTGTCAG	-	-	OMIM***: 603028	PROBSYNTESIS	TÜRKİYE
	TLR2PR	TGTGGAGTGGGAAATCAGGGGACTC	FAM	BHQ-1		PROBSYNTESIS	TÜRKİYE
	TLR4EXP1	CTGGTGGCTGTGGAGACAAATC	-	-		PROBSYNTESIS	TÜRKİYE
TLR4	TLR4EXP2	GTCCAAGTGCTCTAGATTGGTCAG	-	-	OMIM: 603030	PROBSYNTESIS	TÜRKİYE
	TLR4EXPR	CTCTAGAGAACTTCCCCATTGGACATCTC	FAM	BHQ-1		PROBSYNTESIS	TÜRKİYE
BETA ACTIN	AKTYP1	CCTGGCACCCAGCACAATGAAG	-	-		PROBSYNTESIS	TÜRKİYE
(ACTB)	AKTY1P2	GGGTGTAACGCAACTAAGTCATAGTC	-	-	OMIM: 102630	PROBSYNTESIS	TÜRKİYE
	AKT-FAM-PR	AGATCATTGCTCCTCCTGAGCGCAAG	FAM	BHQ-1		PROBSYNTESIS	TÜRKİYE

*Primary probes were designed with **OLIGOYAP 9.0** program

***Online Mendelian Inheritance in Man (OMIM®)
